# Ultrasensitive Detection of Tetracycline Using Boron and Nitrogen Co-Doped Graphene Quantum Dots from Natural Carbon Source as the Paper-Based Nanosensing Probe in Difference Matrices

**DOI:** 10.3390/nano10091883

**Published:** 2020-09-20

**Authors:** Hai Linh Tran, Win Darmanto, Ruey-An Doong

**Affiliations:** 1 Department of Biomedical Engineering and Environmental Sciences, National Tsing Hua University, 101, Sec. 2, Kuang Fu Road, Hsinchu 30013, Taiwan; s106012885@m106.nthu.edu.tw; 2 Department of Biology, Faculty of Science and Technology, Airlangga University, Surabaya 60115, Indonesia; windarmanto@fst.unair.ac.id; 3 Institute of Analytical and Environmental Science, National Tsing Hua University, 101, Sec. 2, Kuang Fu Road, Hsinchu 30013, Taiwan

**Keywords:** boron, nitrogen co-doped graphene quantum dots (B,N-GQDs), passion fruit juice, microwave-assisted, paper strip sensor, tetracycline, human serum

## Abstract

Herein, the boron and nitrogen co-doped 0-dimensional graphene quantum dots (B,N-GQDs) with high quantum yield (QY) were synthesized via microwave-assisted hydrothermal method at 170 °C for 20 min using fresh passion fruit juice and boric acid as the starting materials. The 3–6 layers of B,N-GQDs with mean particle size of 9 ± 1 nm were then used for ultra-sensitive and selective detection of tetracycline in aqueous and biological media. The hybridization of boron and nitrogen atoms into the GQD structures increases the intensity of electronegative, resulting in the enhancement of QY to 50 ± 1%. The B,N-GQDs show their excellent analytical performance on tetracycline determination after 2 min of reaction under an optimal condition at pH 5. The linear range of 0.04–70 µM and with limits of detection (LOD) of 1 nM in phosphate buffer saline (PBS), 1.9 nM in urine and 2.2 nM in human serum are obtained. Moreover, the high selectivity of tetracycline by B,N-GQDs over the other 23 interferences is observed. The π-π interaction and electron donor-acceptor principle play pivotal roles in enhancing the ultra-sensitivity and selectivity of B,N-GQDs toward TC detection. Moreover, the B, N-GQD based paper nanosensor exhibits an excellent analytical performance on visual detection of 0.1–30 µM TC in human serum. Results of this study clearly indicate the feasibility of synthesis of B,N-GQDs derived from passion fruit juice for ultrasensitive tetracycline detection, which can open an avenue to use natural products for the preparation of environmentally benign and biocompatible carbon nanomaterials for highly sensitive detection of drugs, antibiotics, organic compounds and biomarkers.

## 1. Introduction

Tetracycline (TC) is one of the wide-spectrum and efficient antibiotics widely employed to treat and prevent infections caused by bacteria in humans and animals [1]. In addition, TC is a protein synthesis inhibitor widely used in agriculture, aquaculture and animal husbandry due to their low cost and effective antibacterial property [2]. However, the uncontrolled use of antibiotics, particularly in the large-scale animal husbandry, has released large amounts of TC to the environment, leading to the severe danger to human health [3]. Therefore, the search of an eco-friendly and cost-effective tool to accurately detect TC is increasingly demanded.

The synthesis of nanomaterials such as silver [4], gold [5], magnetic nanoparticles [6], V_2_O_5_ [7], organic-metal frameworks [8] and inorganic quantum dots [9] for biological applications has recently received increasing interest. Moreover, carbon dots (CDs) synthesized from natural carbon sources such as fruit juice [10], pine wood [11], vegetable [12], grass [13] and tobacco [14] has been reported to exhibit an excellent optical property for the bio-imaging of microorganisms as well as for the detection of chemicals. Graphene quantum dots (GQDs), a 0-dimensional graphene family fabricated from various carbon sources such as citric acid and glucose, have been demonstrated as a superior fluorescence sensing element for the detection of organic pollutant, heavy metal ions, biological metabolites and cancer markers because of the low toxicity, biocompatibility, easy fabrication and cost effectiveness [11,14,15,16]. A previous study has fabricated the dopamine-functionalized GQDs for effective detection of Fe^3+^ ions [17]. However, the quantum yield (QY) of GQDs is usually not high in comparison with CDs. Therefore, the enhancement of QY of natural product-derived GQDs to increase their sensitivity and selectivity is important.

The doping of GQDs with heteroatoms is an effective strategy to tune the intrinsic electronic band and fluorescence property of graphene-based nanomaterials [18,19,20,21]. Various elements such as N, B, S, Cl, P, N and F have been incorporated into the GQD framework and the heteroatom-doped GQDs consistently show the favorable fluorescence performance [20,22,23] Tran and Doong [24] have prepared S-doped GQDs for ultra-sensitive and selective detection of hemoglobin in human serum. In addition, it has also been reported that co-doping of carbon with two heteroatoms such as B and N can effectively produce more electron active sites than singly doped counterparts, leading to the complementary coupling effects among heteroatoms [25]. Therefore, doping of B and N atoms into a graphene framework can produce chemical disturbance and subsequently results in the unique optical characteristics for nanosensing applications [26]. Moreover, GQDs synthesized from different carbon and doping sources may lead to the different microstructural and physicochemical properties such as morphology, shape, size, optics and defects. Although several carbon-based materials have been developed for optical sensing of tetracycline [27,28], no work is attempted to fabricate B, N co-doped GQDs for the detection of TC in different media. In addition, the use of passion fruit juice as the environmentally benign material for the green synthesis of GQDs via microwave system has not been reported yet. 

**Scheme 1:**
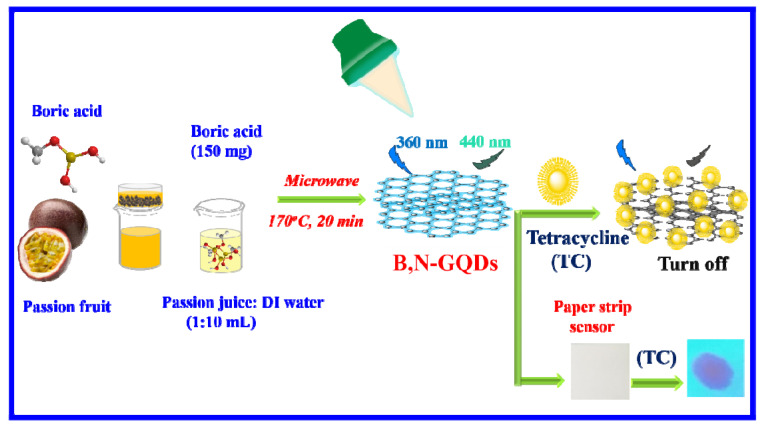
The environmentally benign fabrication of B,N co-doped graphene quantum dots (B,N-GQDs) by microwave-assisted hydrothermal method using passion fruit juice and boron acid as the starting materials and the application to tetracycline detection in PBS (phosphate-buffered saline) and human serum.

Herein, B,N co-doped GQDs (B,N-GQDs) have been fabricated using the microwave-assisted hydrothermal method using passion fruit juice and boric acid as the starting materials and then used as the label-free fluorescent probe for TC determination in phosphate-buffered saline (PBS) and human serum. As shown in Scheme 1, the blue fluorescent B,N-GQDs was hydrothermally fabricated at 170 °C for 20 min. After dialysis to remove impurities and un-reacted reagents, a blue fluorescence at 440 nm is observed when B,N-GQDs are irradiated with UV light. The transmission electronic microscopic (TEM) results show that 5–14 nm B,N-GQDs with a mean particle size of 9 ± 1 nm and a high QY of 50 ± 1% are obtained. The B,N-GQDs exhibit a superior analytical performance on TC detection after 2 min of reaction under an optimal condition at pH 5. A linear range of 3 orders of magnitude with a limit of detection (LOD) value of 1 nM in PBS, 1.9 nM in urine and 2.2 nM in human serum are achieved. The selection of passion fruit as the raw carbon source is mainly attributed to the fact that the passion fruit is a naturally occurring product and the relatively abundant nitrogen in passion fruit juice can serve as the N-doping source, which can reduce the usage of chemical to partially fulfill the green chemistry and circular economy. Moreover, the selectivity of TC by B,N-GQDs over the other 23 interferences in human serum is examined. To the best of our knowledge, this is the first study on utilizing passion fruit juice as the carbon sources for green synthesis of B,N-GQDs as the label-free fluorescent probe for effectively detection of antibiotics in different media, which can open a gateway in design a simple, cost-benefit and environmentally friendly carbon-based materials for a wide variety of practical applications in green chemistry, monitoring, analysis and biomedical engineering.

## 2. Experimental

### 2.1. Chemicals and Materials

Boric acid (H_3_BO_3_) was obtained from Riedel-de Haën (Seelze, Germany). Sodium hydroxide (NaOH) and tetracycline (TC) were obtained from Sigma-Aldrich (Milwaukee, WI, USA). Passion fruit (*Passiflora edulis L.*), used as a green carbon source, was purchased from different local markets, Hsinchu, Taiwan. Commercially filter-sterilized human serum was bought from Gemini bio-product (West Sacramento, CA, USA). The dialysis bag (1k Da) was obtained from Membrane Filtration Products, Inc. (Seguin, TX, USA). All the other reagents were used as received without further treatment. Moreover, bi-distilled deionized water (DI water, 18.2 MΩ cm) was used throughout the experimental course.

### 2.2. Synthesis of B,N-GQDs

The B,N-GQDs were fabricated via microwave treatment at 170 °C for 20 min using passion fruit juice as a green carbon source and boric acid as the doping precursor. Practically, seeds in fresh passion fruit was removed and then the juice was centrifuge at 11,000 rpm for 10 min to remove debris. The suspended fibers contained in the juice was removed by a 0.22 μm membrane filter. Subsequently, the solution of passion juice was diluted 10 times by DI water. 150 mg boric acid and 20 mg NaOH were added into 10 mL of diluted fresh juice and then sonicated for 5 min. It is noteworthy that the pH of passion fruit juice was measured to be 3.3–3.6. Therefore, NaOH was used in this study to adjust the pH of solution as well as to serve as the basic catalyst to magnify the formation of B, N-GQDs in microwave-assisted hydrothermal method. Afterward, 10 mL of the mixed solution was transferred into a microwave vessel and heated at a constant temperature of 170 °C for 20 min at 800 W to carbonize the passion fruit juice. After hydrothermal treatment, the sample was cooled to room temperature and the color of solution turned into brown. The B,N-GQDs in supernatant was then harvested by centrifugation at 11,000 rpm first and then dialyzing in a 1 kDa dialysis bag for 24 h using DI water as the dialysate.

### 2.3. Surface Characterization

The chemical species of B,N-GQDs were characterized with an ESCA Ulvac-PHI 1600 X-ray photoelectron spectrometer (XPS, Kanagawa, Japan) with Al Kα radiation at 1486.6 ± 0.2 eV as the photon energy. The crystallinity of B,N-GQD was recorded with a Bruker D8 X-ray diffractometer (XRD) (Bremen, Germany) with Cu Kα radiation (λ = 1.5406 Å) in the 2θ range of 10°–50°. Raman spectrum of B,N-GQDs was acquired with a Burker Senterra micro-Raman spectrometer (Bremen, Germany). Horiba FT-720 spectrophotometer (Minami-ku Kyoto, Japan) was used to record the FTIR spectra of B,N-GQDs. TEM (JEM-ARM200F, TEM, Jeol, Tokyo, Japan) and high-resolution TEM (HRTEM, JEM-2010, Peabody, MA, USA) were used to examine the morphology and size of B,N-GQDs at an accelerating voltage of 300 kV. A JEOL JSM-7610F high-resolution scanning electron microscope (HRSEM, Peabody, MA, USA) was used to identify the elemental distribution patterns of B,N-GQDs. The topographical information and thickness of B,N-GQDs were acquired using an Agilent 5500 atomic force microscope (AFM, Santa Clara, CA, USA). The UV-visible absorption spectra of B,N-GQDs were recorded using Hitachi U-4100 UV-VIS-NIR spectrophotometer (Tokyo, Japan). The fluorescence spectra were determined with Hitachi F-7000 fluorescence spectrophotometer (Tokyo, Japan). Moreover, the elemental contents including C, H, N and S in passion fruit juice was analyzed by an Elemental Analyzer (Elementar Vario EL cube, Langenselbold, Germany).

### 2.4. Detection of TC by B,N-GQDs in PBS, Urine and Human Serum

The stock solution of TC was prepared by adding 4.44 mg TC in 1 mL of PBS at pH 5. In addition, 3 mL of 15 μg mL^−1^ B,N-GQD solutions were prepared in PBS and then the stock TC solution was added at an appropriate amount to get the desired concentration ranging from 0.04 to 70 μM. The detection of TC was performed by recoding the optical property of TC solutions after 2 min of incubation at emission wavelength of 440 nm after the excitation of 360 nm UV light. The selectivity of TC by B,N-GQDs was also examined by fluorescence quenching over 23 interferences. Moreover, the urine and human serum studies were performed according to the guidelines and regulations of the ethics committee and the IRB of National Tsing Hua University (IRB No. 10802HM004).

### 2.5. B,N-GQD Based Paper Strip Nanosensor for TC Detection in Human Serum

The B,N-GQD based paper-strip sensor was fabricated originally from the cellulose filter paper with a pore size of 25 μm and a diameter of 125 mm. A sheet of cellulose filter paper was cut into small strips with area of 0.5 × 0.5 cm^2^. The paper-strips were then immersed into 1 mL of 0.5 mg mL^−1^ B,N-GQD solution for 5 min. Finally, the B,N-GQD based paper strip sensor were fabricated by drying in an oven at 60 °C. 

For visual detection of TC in human serum, the 0.1–30 µM TC solutions were prepared in 10× diluted human serum to create a matrix environment for sensing probe. The sensing ability of the paper-strips sensor was evaluated by adding 1 µL of various concentrations of TC solution into the center of paper-strips sensor. The change in color on paper strip sensors was visually detected under UV light irradiation and the images were recorded by a smartphone.

### 2.6. Cytotoxicity Assay of B,N-GQDs

The MTT assay was used to investigate the cytotoxicity of various concentrations of N-GQDs and B,N co-doped GQDs at 0, 100, 200, 400 and 600 μg mL^−1^ using 3-(4,5-dimethylthiazol-2-yl)-2,5-diphenyltetrazolium bromide (MTT) as the standard reagent. The MCF-7 cells were incubated in a 96-well plate containing 3 × 10^2^ cells and then 0–600 μg mL^−1^ N-GQDs and B,N co-doped GQDs were added to the culture media and incubated for 24 h at 37 °C in an atmosphere of 5% CO_2_. After 24 h of incubation, the 96-well plate was washed with 0.1 M UPSB at pH 7.4 and then the MCF-7 cells were kept in an incubator for 48 h. The cell viability of each well was determined by the absorbance using microplate reader at 570 and 600 nm.

## 3. Results and discussion

### 3.1. Characterization of B,N-GQDs

**Figure 1:**
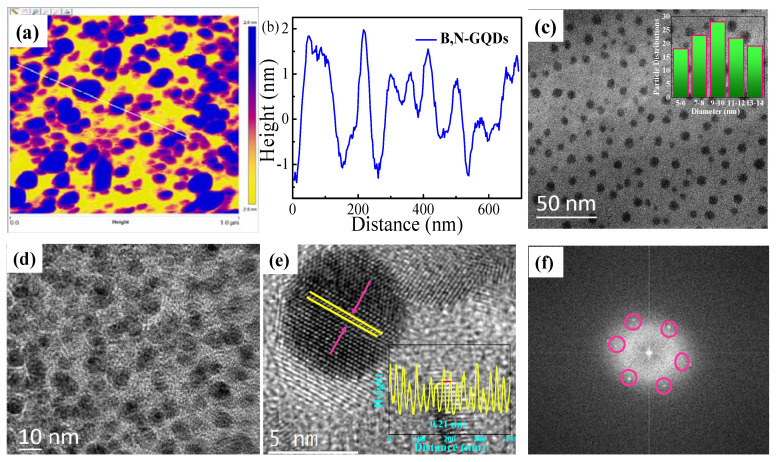
(**a**) The atomic force microscopy (AFM) image with inset showing the height distribution of B-N-GQDs, (**b**) 3D morphology of B,N-GQDs on the Si substrate, (**c**) low and (**d**) high magnification of transmission electron microscopy (TEM) images, (**e**) high resolution TEM (HRTEM) image and (**f**) fast Fourier transform (FFT) analysis pattern of the as-prepared B,N-GQDs. The inset of Figure 1c is the particle size distribution of as-prepared B,N-GQDs.

AFM was first used to examine the topography of B, N-GQDs. As illustrated in Figure 1a, the AFM image displays the good dispersivity of B,N-GQDs on the Si substrate, in which blue spots are B,N-GQDs by their shape. The inset image in Figure 1a,b show the corresponding three-dimensional plots of Figure 1a and the height distribution of B,N-GQDs is 0.9–2 nm, indicating that the B,N-GQDs are 3–6 layers in thickness [29]. It is noteworthy that GQDs are one kind of carbon dots (CDs), which the height is usually less than 10 layers and the conductivity is good. In this study, some CDs may exist during the fabrication using passion fruit as the raw materials. However, most carbon-based quantum dots obtained in this study are B,N-doped GQDs. In addition, the morphology of B,N-GQDs was also carried out by electronic microscopy. The TEM images (Figure 1c,d) evidence that the particle size distribution of B,N-GQDs is narrow, which is around 5–14 nm with an average diameter of 9 ± 1 nm (Inset of Figure 1c). The inter-lattice spacing shown in the HRTEM image is 0.21 nm (Figure 1e), which is the typical (100) plane of defect-free graphene [30]. Moreover, the 6 white dots in hexagonal arrangement, denoted in pink circles in Figure 1f, is clearly observed, which indicates that B,N-GQDs belong to the benzene ring carbon atoms.

The carbon structures of B,N-GQDs was further identified by Raman spectroscopy (Figure S1a,b, Supplementary materials). Two characteristic peaks occur at 1362 and 1584 cm^−1^, which signify the presence of sp^3^ defects (D band) and the in-plane vibration of sp^2^ carbon (G band), respectively. The broader peak of D band indicates the intercalation of B and N atoms into the conjugated carbon backbone of GQDs, which leads to a slightly disordered structure of the B,N-GQDs [31,32]. Moreover, the ratio of D and G band intensity (I_D_/I_G_) is 0.89, which represents the crystalline and graphitic nature of B,N-GQDs [33]. The XRD patterns of B,N-GQDs also show a broad peak at 2θ of 26.0° and a small peak at 2θ of 40.3°, which are the characteristic peak of (002) interlayer spacing and (100) in-plane lattice spacing of graphene in B,N-GQDs structure (Figure S1c, Supplementary materials) [34].

To further evaluate the distribution patterns of elements in B,N-GQDs, energy dispersive spectroscopy (EDS) and elemental mapping of elements were investigated. As shown in HRSEM image (Figure S2, Supplementary materials), a spherical shape of B,N-GQDs is observed and the EDS spectrum indicates that the C, O, B and N ratios are 40.0, 13.5, 18.3 and 25.2 at%, respectively, which confirm that boron and nitrogen are successfully decorated on graphene through the microwave method. It is noteworthy that a relatively high content of N is observed in the EDS spectrum, presumably attributed to the inherent component in passion fruit juice. The elemental analysis illustrated in Table S1 (Supplementary materials) indicates that the weight percentages of O, C, H, N and S in pure passion fruit juice are 54.1, 38.3, 6.6, 0.75 and 0.28 wt%, respectively, clearly depicting that N element is an original ingredient in passion fruit. This result also means that B,N-GQDs contain both doped B atoms and inherent N element, which may enhance the quantum yield of B,N-GQDs for optical applications.

**Figure 2:**
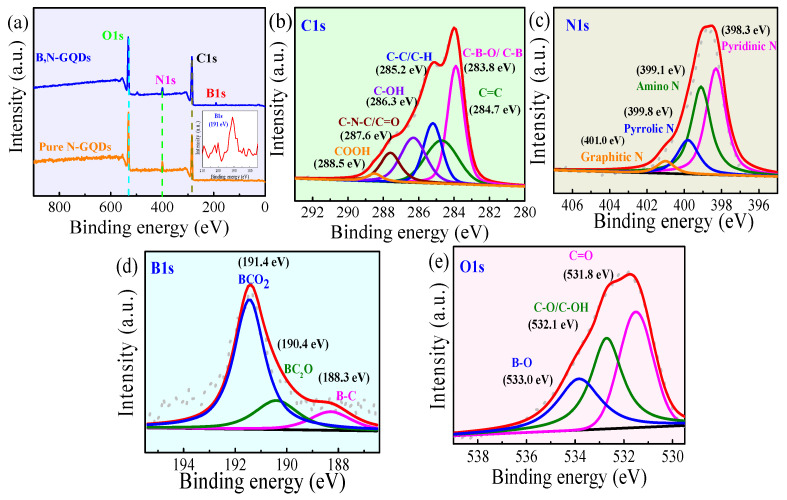
The (**a**) X-ray photoelectron spectroscopy (XPS) survey scan of pure N-GQDs and B,N-GQDs and deconvoluted (**b**) C 1s, (**c**) N 1s, (**d**) B1s, (**e**) O 1s peaks of B,N-GQD.

The XPS was employed to gain further structural insight and to verify the existence of N and B species on B,N-GQDs. Figure 2a displays the XPS survey scans of as-prepared pure N-GQDs and B,N-GQDs. The XPS spectra of both N-GQDs and B,N-GQDs contain C 1s, N 1s and O 1s located at 284, 400 and 532 eV, respectively [7,24,35]. It is also noted that a small peak centered at 191 eV, which belongs to B 1s peak, can be found in the survey scan of B,N-GQDs, proving again the successful co-doping of B, N into the GQD carbon lattice [36]. The C, O, N and B contents in B,N-GQDs are calculated to be 59.1, 32.3, 6.2 and 2.4 wt% (Table S2, Supplementary materials). Furthermore, the deconvoluted C 1s spectrum (Figure 2b) shows the C=C, C–C/C–H, C–OH and COOH groups at 284.7, 285.2, 286.3 and 288.5 eV, respectively [7,17,33]. The prominent peaks at 283.8 eV and 287.6 eV are the C–B/C–B–O and C–N–C/C=O groups, which proves that B, N elements are successfully doped into GQDs network. Furthermore, the N 1s spectrum of Figure 2c is deconvoluted into 4 peaks at 398.83 (pyridinic N), 399.1 (amino N), 399.8 (pyrrolic N) and 401.0 eV (graphitic N). In addition, three peaks shown in the deconvoluted B 1s peak at 188.3, 190.4 and 191.4 eV are ascribed as the B–C, BC_2_O and BCO_2_ bonds on B,N-GQD (Figure 2d) [25,36,37]. For O 1s species, the deconvoluted spectrum also illustrates three peaks at 533.0 (B–O), 532.1 (C–O/C–OH) and 531.8 (COOH) eV [36,37,38], Moreover, after the modification of B atom the deconvoluted C 1s and O1s spectrum show a shift peak to high binding energy (Figure 2b,e, respectively) compared to that for the C 1s and O 1s spectrum of pure N-GQDs (Figure S3a,b). It means that upon doping of B into the GQDs structure, it combined with the surface functional groups as well, confirming the presence of the co-doping of N and B on GQD networks (Figure 2e). 

### 3.2. Optical Property of B,N-GQDs

The UV-visible spectra of B,N-GQDs, TC and B,N-GQDs/TC mixture were also determined. As illustrated in Figure S4 (Supplementary materials), the small absorption shoulder of B,N-GQDs at 270 nm is the π-π* transition of aromatic sp^2^ domains and the broad peak at 330 nm is the n-π* absorption bands of electrons in the C=O bond [17,39] In addition, pure TC shows absorption at 278 and 362 nm, which display the π-π and n-π* transition of TC molecule, respectively. The interaction between B,N-GQDs and TC leads to the shift in absorbance peak. After adding 10 μM TC to B,N-GQDs, the absorption peak at 330 nm disappears and the TC peak at 362 nm shifts to 385 nm, primarily due to the adsorption of TC onto the B,N-GQDs surface.

**Figure 3:**
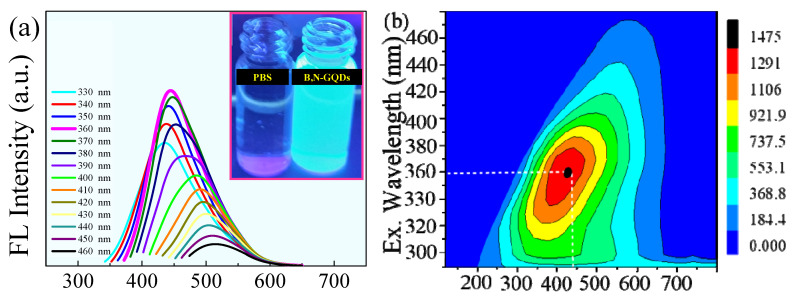
(**a**) The fluorescence spectra of B,N-GQDs irradiated with 330–470 nm UV-visible light (Inset is the photo of B,N-GQDs and PBS under 365-nm UV light irradiation) and (**b**) 2D excitation-emission contour map of B,N-GQDs.

Figure 3a displays the fluorescence spectra of B,N-GQDs irradiated at 330–460 nm. The emission bands centered at 430–520 nm are clearly observed. The emission intensity increases as the excitation wavelength increases from 330 to 360 nm and then decrease 460 nm, the highest emission peak occurs at 440 nm when B,N-GQDs is excited at 360 nm. The inset of Figure 3a clearly shows that the color of B,N-GQDs solution turns into strong blue light, whereas the PBS is still transparent after the 365 nm UV light irradiation. Moreover, the intensity of emission fluorescence peak decreases and red-shifts from 420 to 520 nm when the excitation wavelength changes to 330–460 nm, depicting that the excitation wavelength of 360 nm is optimal to produce fluorescence intensity for TC detection. The photo in the inset of Figure 3b shows the 2-dimensional (2D) excitation-emission contour maps of the as-prepared B,N-GQDs. The fluorescence intensity contour is highly dependent on the excitation wavelength. Interestingly, the highest fluorescence intensity, located in the black region of contour mapping, appears at 440 nm when 360 nm is used to irradiate B, N-GQDs. Figure S5 (Supplementary materials) shows the change in fluorescence of B,N-GQDs in PBS in the absence and the presence of 30 µM TC under 365-nm UV and visible light irradiation. As illustrated in Figure S5a, all the solutions are colorless in the presence of visible light. When 30 µM TC is added to the bottle containing B,N-GQDs, a change in color to yellow in comparison with PBS solution and B,N-GQDs is observed (Figure S5b). In contrast, a strong blue fluorescence of B,N-GQD solution appears, while the buffer solution is still transparent in the presence of UV light (Figure S5c). Interesting, the fluoresce intensity quenches dramatically when TC is added. This result clearly indicates that B,N-GQDs can produce a strong blue fluorescence after excitation, while TC is an effective quencher to decrease the fluoresce intensity of B,N-GQDs.

The QY of carbon-based nanomaterials can affect the sensitivity of sensing material. Usually, the QY of GQDs is relatively low because of the few layers of graphene structures [14]. Therefore, the QYs of N-GQDs and B,N co-doped GQDs were determined using the standard quinine sulfate solution (*Φ* = 0.54) in 0.1 M H_2_SO_4_ as the reference. The QYs of the N-GQDs and B,N-GQDs using passion fruit juice as the carbon source are calculated to be 27% and 50 ± 1%, respectively (Figure S6, Supplementary materials), which is higher than those reported carbon-based quantum dots fabricated from the natural products including aloe, bamboo tar and gelatin (5.2–34%) [11,40] (Table S3, Supplementary materials).

The B,N-GQDs show a more pronounced electron affinity than that of N-GQDs despite the low electronegativity of B elements (2.58) compared to that of N atoms. Moreover, the oxygen bridges in B,N-GQDs (B–O, C–O and C=O) can enhance the electron poverty in carbon atoms because of the high electronegativity of O atoms. Therefore, the resulting QY of B,N-GQDs is higher than that of N-GQD because N atoms are inherently inside the passion fruit juice [26]. Furthermore, boron atoms in carbon structures can function as electron acceptors and thus redistribute the *π* electrons in the carbon sp^2^ structure to deprive the Fermi level as well as to enhance the electron affinity [25,41].

**Figure 4:**
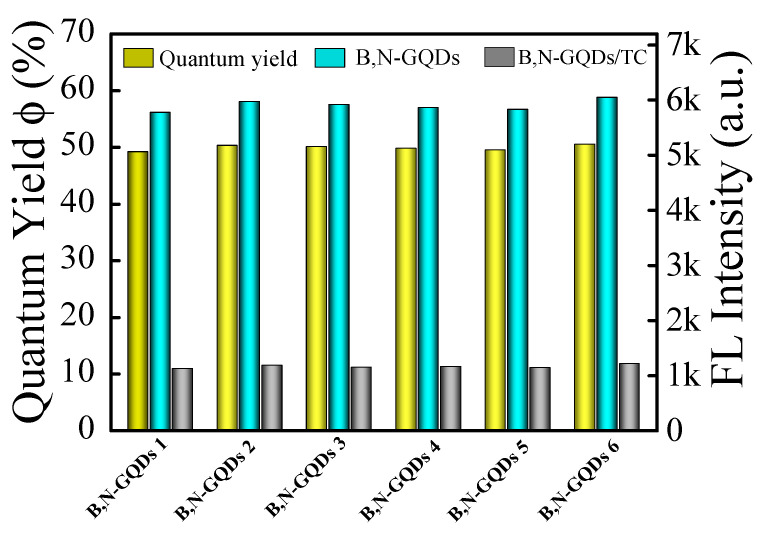
Comparison of the percentage quantum yield, the fluorescence intensity of B,N-GQDs was synthesized from different passion fruit juice harvested from different sources at different times of the year and after added 30 µM TC.

In addition, the QY as well as fluorescence intensity of B,N-GQDs fabricated from 6 different sources including local and imported passion fruits are examined to understand the variation of sensing probes from different natural products. As shown in Figure 4, the QYs and the fluorescence intensities of these six different B,N-GQDs are in the range of 49–51% (yellow columns), while the fluorescence intensities range between 5785 and 6048 a.u. (cyan columns). After the addition of 30 μM TC solutions into B,N-GQDs solutions, the fluorescence intensity of B,N-GQDs decreases dramatically and only fluorescence intensity range of 1118–1205 a.u. (gray columns) is observed. This result clearly demonstrates the sensing probe of B,N-GQDs is stable and reliable when prepared with different natural products sources, which can be used as the nanosensing probe for detection of TC. 

### 3.3. Detection of TC By B,N-GQDs

**Figure 5:**
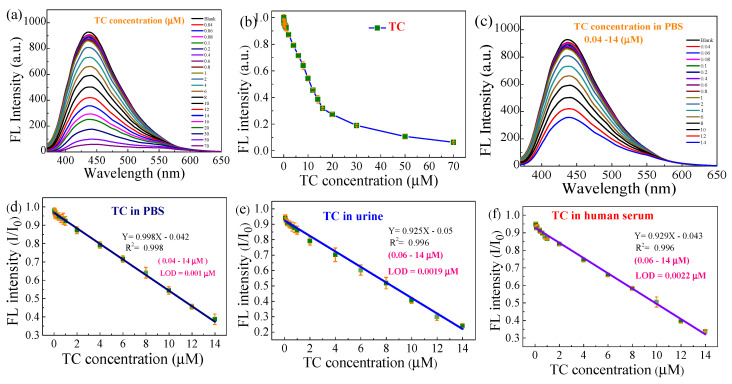
(**a**) Change in fluorescence spectra of B,N-GQDs in the presence of 0–70 μM TC in PBS, (**b**) The fluorescence intensity ratio as a function of TC concentration, (**c**) The fluorescence spectra of 0–14 µM TC by B,N-GQDS as the fluorescence sensing probe in PBS and the linear calibration curves for the detection of TC by B,N-GQDs in (**d**) PBS, (**e**) urine and (**f**) human serum, respectively.

Since B,N-GQDs have the superior optical property which can serve as the label-free nanosensing probe, the applicability of B,N-GQDs was evaluated with the addition of TC. Figure 5a displays the change in fluorescence intensity of B,N-GQDs after the addition of 0–70 μM TC solutions. The fluorescence intensity of B,N-GQDs at 440 nm decreases upon increasing TC concentration. It is interesting to note that the fluorescence peak at 440 nm remains unchanged at all the tested concentrations, which depicts the stable fluorescence property of B,N-GQDs for sensing purposes. Figure 5b shows the change in fluorescence intensity ratio (I/I_0_) as a function of TC concentration. The fluorescence intensity ratio decreases rapidly from 40 nM to 14 µM and then follows a slight decrease in I/I_0_ at a high TC concentration of 14–70 µM. The calibration curve follows a two-phase linear relationship, which is in good accordance with the previously reported results using doped GQDs for the detection of a wide variety of analytes in aqueous media [14,23,30]. This result also indicates that B,N-GQDs can effectively detect a wide concentration range of TC.

The B,N-GQDs spectra for TC detection in PBS using B,N-GQDs as the label-free fluorescence probe exhibit a wide linear range from 0.04 to 14 μM (Figure 5c). Moreover, the B,N-GQDs spectra for low concentration of TC detection (0.04 to 2 μM) in PBS, urine and human serum samples also illustrate in Figure S7a–c. The calibration curve of TC in PBS by B,N-GQDs shows a good correlation coefficient (*r^2^*) of 0.998. The LOD, which can be determined by 3σL where σ is the relative standard deviation for 7 replicates and L is the lowest TC concentration added, is calculated to be 1 nM in PBS (Figure 5d and Figure S7a). To further investigate the performance of B,N-GQDs nanosensor for TC detection in real samples, the detection of TC in human urine and serum are also examined. For human urine, B,N-GQDs nanosensor displays a wide linear detection range of 0.06–14 μM in 10x diluted in human urine sample with a good correlation coefficient (*r^2^*) of 0.996. An excellent LOD of 1.9 nM in urine sample is also obtained (Figure 5e and Figure S7b). As illustrated in Figure 5f, a good linear relationship in the TC concentration range of 0.06–14 μM with *r^2^* of 0.996 in 10× diluted human serum is observed. In addition, the LOD for TC detection is 2.2 nM in human serum (Figure S7c). This performance is significantly superior to those most reported data using different nanomaterials for TC detection summarized in Table 1. 

**Figure 6:**
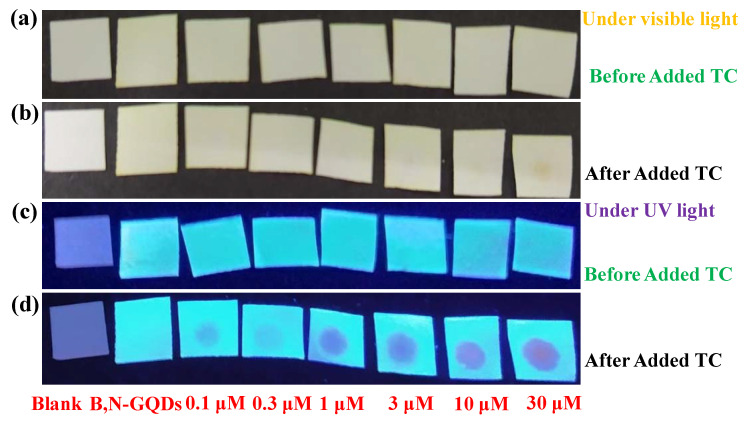
The visual detection of TC using B,N-GQD decorated paper sensor. Photos of pure cellulose paper and B, N co-doped GQD-based cellulose paper in the (**a**) absence and (**b**) presence of TC under visible light irradiation, (**c**) The color of B,N co-doped GQD-based paper sensor in the absence of TC under UV light irradiation and (**d**) the change in color of B,N-GQD decorated paper sensors after the addition of 0.1–30 μM TC under 365 nm UV light irradiation.

After the successful detection of TC in aqueous and biological media, the applicability of using B,N-GQD-based paper-strip nanosensor was then examined in 10× diluted human serum. Figure 6 displays the photograph of the B,N-GQDs paper-based nanosensors for the visual detection of various concentrations of TC from 0.1 to 30 μM under visible light and UV light. It is clear that the B, N-GQD-based cellulose paper in the absence and presence of TC shows no fluorescence property under visible light irradiation (Figure 6a,b). However, the paper strip emits a strong blue fluorescence light under UV light irradiation (Figure 6c) and the fluorescence of paper-strip sensor quenches rapidly after the addition of 1 μL of 0.01–30 μM TC onto the paper strip nanosensors (Figure 6d). This results is in good agreement with the photo images of B,N-GQD solution (Figure S5), signifying that B,N-GQDs can be firmly attached onto the hydrophilic cellulose base paper. Moreover, the fluorescence intensity decreases with the increase in TC concentration, obviously indicating that the paper-based nanosensor is applicable for the rapid visual detection of TC in human serum samples. In short, we have designed a simple, inexpensive, disposable and eco-friendly paper strip tool for the rapid screening of TC in human serum samples.

### 3.4. Selectivity of B,N-GQDs

**Figure 7:**
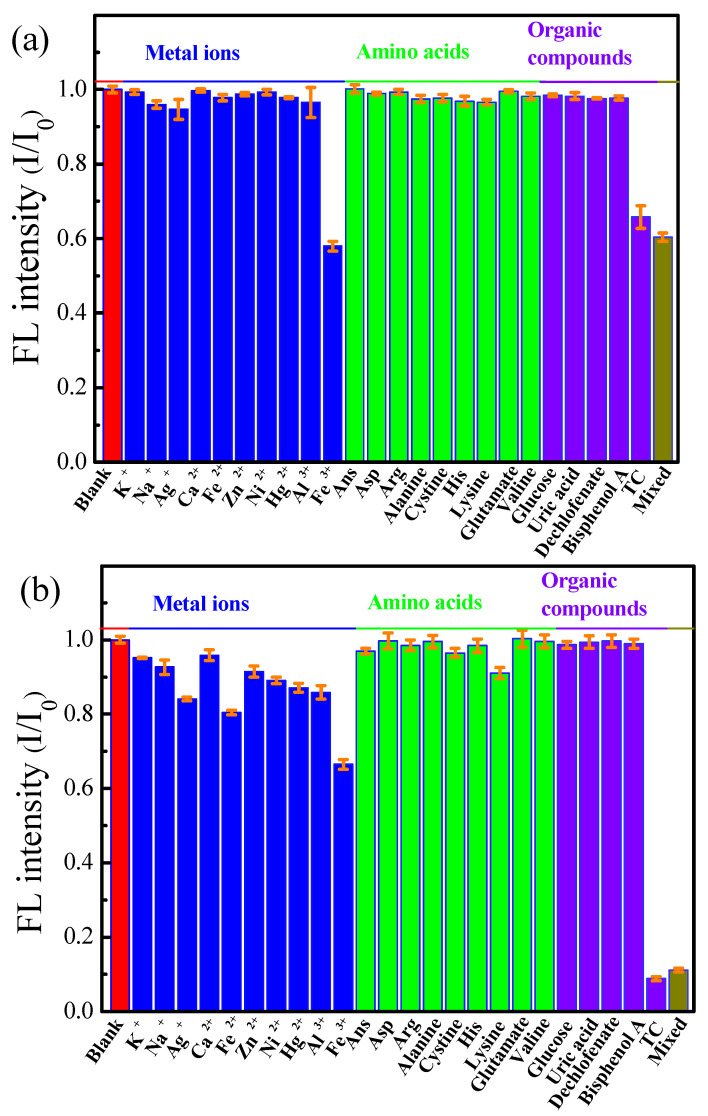
The selectivity of TC by (**a**) N-GQDs and (**b**) B,N-GQDs over the other 23 interfering chemicals. The concentration used for all the chemicals used is 50 µM.

The selectivity of sensing system is always crucial for the successful application to real samples. In this study, the selectivity of B,N-GQDs was further examined by the addition of another 23 interfering molecules. A broad spectrum of interferences including cations, amino acids, organic compounds and other antibiotics is selected based on the possible inorganic and biological components in human serum. Figure 7 shows the ratio of fluorescence intensity (I/I_0_) of undoped and B,N co-doped GQDs in the presence of TC and other 23 different interfering molecules in PBS. The concentrations used in this study are all 50 µM and the fluorescence spectra of GQDs and B,N-GQDs are recorded after 2 min of reaction. The pure N-GQDs show less sensitive (I/I_0_ = 0.92–0.997) toward most interference species detection (Figure 7a). However, the addition of Fe^3+^ displays an obvious decrease in I/I_0_ ratio (0.58), which is similar to that of TC. Several studies have demonstrated the excellent sensitivity of GQD toward Fe^3+^ detection [11,17], which is similar to the result obtained in this study. However, the B,N co-doped GQD shows the excellent selectivity toward TC detection. As displayed in Figure 7b, addition of 50 µM interferences has less influence on the fluorescence intensity of B,N-GQDs. Although the addition of some ions like Ag^+^, Fe^2+^ and Fe^3+^ show slight decrease in I/I_0_ to 0.68–0.80, the fluorescence intensity ratio is still much higher than that of TC (0.13). After mixing all the interfering molecules with TC, the I/I_0_ ratio (0.147) is almost similar to the individual TC, depicting the superior selectivity of B,N-GQDs toward TC detection.

### 3.5. Possible Sensing Mechanism for TC Detection by B, N-GQDs

The pH plays a pivot role in affecting the fluorescence intensity of sensing probe. Figure S8 (Supplementary materials) shows that the fluorescence intensity of B,N-GQDs in 0.1 M PBS increases under acidic condition at pH 3–5 and then decreases when the pH increases from 6 to 10. Moreover, the fluorescence intensity quenches dramatically after the addition of 30 µM TC. The I/I_0_ of B,N-GQD in the presence of TC decrease from 0.254 at pH 3 to 0.146 at pH 5 and then gradually increases to 0.296 at pH 10, depicting the excellent quenching effect of TC on the fluorescence of B,N-GQDs at pH 5. It is noteworthy that the triprotonic acid and the dissociation constants (pKa) of tetracycline are 3.3, 7.7 and 9.7 [48], which means that TC will be deprotonated under the acid condition of pH 5. Since B atoms in GQD structures can serve as electron acceptor, the deprotonated TC acts as an electron donor to adsorb onto B,N-GQD surface, resulting in the significant quenching of the fluorescence produced from GQDs. At high pH of 7–10, however, the more negatively charged TC would produce repulsive force with the negatively charged B,N-GQDs (Table S4, Supplementary materials) and subsequently leads to the decrease in fluorescence intensity. Therefore, pH 5 is optimal for B,N-GQDs to detect TC.

From the results of selectivity and pH effect on TC detection by B,N-GQD, we can propose the possible detection mechanism of label-free B,N-GQDs fluorescence probe for the turn off-on detection of TC at pH 5. It is noteworthy that both pure N-GQDs and B,N-GQDs have selectivity toward TC detection and B,N-GQDs exhibit superior selectivity toward TC detection in comparison with pure N-GQDs, Therefore, the sensing mechanism is mainly based on the π-π interaction and electron donor-acceptor principle between TC and B,N-GQDs. The doping of B and N elements into carbon structure of GQDs leads to an increase in electronegative intensity to polarize oxygen atoms at the edges of GQDs [25,26,49,50], resulting in the negatively charged of B,N-GQDs. It is also noted that boron can serve as electron acceptor to attract the negatively charged TC at pH 5 by the electron donor-acceptor interaction [51,52], resulting in the quench of the fluorescence intensity at pH 5. However, the increase in pH value would produce more negatively charged TC, which can produce strong repulsion with the negatively charged B,N-GQDs. 

**Figure 8:**
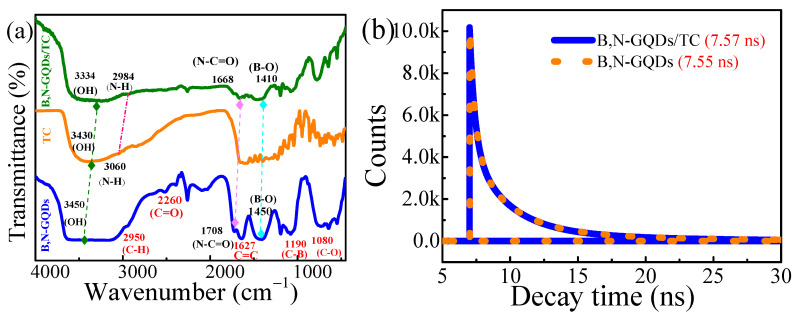
(**a**) The FTIR spectra of B,N-GQDs before and after the addition of TC plus TC alone and (**b**) fluorescence decay time of B,N-GQDs before and after the addition of TC.

Figure 8a displays the FTIR spectra of B,N-GQDs before and after the addition of TC. The as-prepared B,N-GQDs spectrum exhibits peaks at 2950 and 1627 cm^−1^, which can be considered as an indication of the C–H and C=C groups in graphene structure [31,37]. A broad peak at 3450 cm^−1^ is the characteristic OH bond. In addition, peaks at 2260 and 1080 cm^−1^ can be assigned as the C=O and C–O functional groups, which is the characteristic functional groups of carboxylic groups [30,40]. The presence of –OH and –COOH groups means that the B,N-GQD surface is hydrophilic. Moreover, peaks at 1708, 1450 and 1190 cm^−1^ belong to the stretching vibration of N–C=O, B–O and C–B, respectively [33,40]. The appearance of new bonding of B–O (1450 cm^−1^) and C–B (1190 cm^−1^) in comparison with pure N-GQDs signifies the successfully B doped into GQDs network, confirming the doping of B, N on graphene structure. 

The change in functional groups of B,N-GQDs after the reaction of TC is also evaluated. It is clear that most absorption peaks of B,N-GQDs have no obvious change. However, the absorption peaks of OH, N–C=O and B-O stretching vibrations shift to the low wavenumber region, that is, from 3450 to 3334 cm^−1^ for OH, from 1708 to 1668 cm^−1^ for N–C=O and from 1450 to 1410 cm^−1^ for B–O. Furthermore, the absorption peaks of O–H and N–H groups in TC also shift from 3450 to 3334 cm^−1^. A new small bond at 2984 cm^−1^ appears, which is the typical N–H group from TC. These results clearly indicate that the –OH, –NH_2_, –COOH groups on TC structure is attached on B,N-GQDs structure, leading to the shift in wavenumber on FTIR spectra. In addition, the life time fluorescence decay experiments were performed and found that the fluorescence lifetime of pure B,N-GQDs is 7.57 ns (Figure 8b). After the addition of 70 μM of TC to the B,N-GQDs solution, the fluorescence lifetime value of 7.55 ns was observed. The little difference in fluorescence lifetime before and after the addition of TC corroborates that the static quenching between B,N-GQDs and TC is responsible for the quenching mechanism for TC detection [53]. It should be noteworthy that both XPS and FTIR spectral results display the formation of new chemical bonds between B elements and functional groups of GQDs, clearly elaborating that the doping is the surface modification of GQDs. Since B,N-GQDs need to adsorb TC onto the surface before detection [7,29,35], the surface modification of GQDs by doping is sufficient for optical sensing of TC.

### 3.6. Cytotoxicity of GQDs Based Nanomaterials

**Figure 9:**
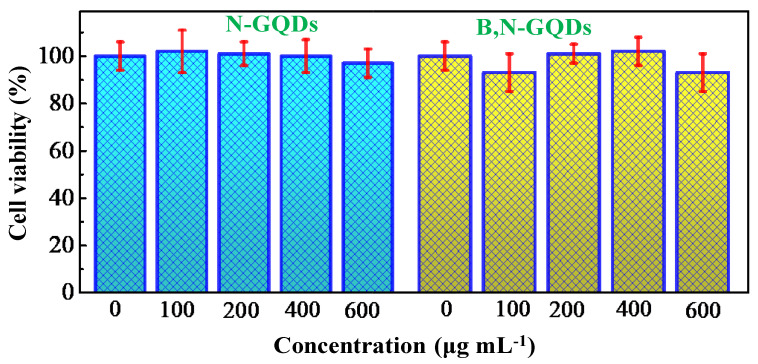
3-(4,5-dimethylthiazol-2-yl)-2,5-diphenyltetrazolium bromide (MTT) assay for the cytotoxicity of MCF-7 cells in the presence of 0–600 µg mL^−1^ pure N-GQDs and B,N co-doped GQDs in human serum samples.

The biocompatibility of N-GQDs and B,N co-doped GQDs was examined using MTT. Figure 9 displays the in vitro cytotoxicity assay of MCF-7 cells in the presence of 0–600 µg mL^−1^ pure and B, N co-doped GQDs. The viability of the MCF-7 cells can be maintained at 97–102% and 93–102% for N-GQDs and B,N-GQDs, respectively, clearly indicating that 100–600 µg mL^−1^ B,N-GQDs have a little cytotoxic effect on MCF-7 in comparison with the control blank. It is noteworthy that no obvious difference in cytotoxic effect between N-GQDs and B,N co-doped GQDs is observed, clearly showing that passion fruit juice is an environmentally benign and biocompatible carbon source, which can fabricate high performance GQDs for sensing of chemicals such as amino acid, protein, molecules in biological samples as well as for living cells applications like bio-imaging and drug delivery.

## 4. Conclusions

In this study, the 5–14 nm B,N-GQDs derived from natural passion fruit juice via microwave treatment have been successfully fabricated and then are used as the label-free fluorescent probe for the ultra-sensitive and selective detection of TC at pH 5. The average size of B,N-GQDs is 9 ± 1 nm with thickness of 0.9–2 nm, which corresponds to 3–6 layers of graphene. The doping of 2.4 wt% B and 6.2 wt% N elements in GQDs can not only boost the emission of blue fluorescence at 440 nm with high QY of 50 ± 1% but also strengthen the electron density of B,N-GQDs to enhance the analytical performance toward TC detection at pH 5. The B,N-GQDs exhibit an excellent nanosensing ability toward TC detection in the concentration range of 0.04–70 µM. The LOD for TC detection is 1 nM in PBS, 1.9 nM in urine and 2.2 nM in human serum. In addition, a superior selectivity of B,N-GQD for TC detection over other 23 interferences is observed, primarily attributed to the π-π interaction and electron donor-acceptor principle. The B,N-GQD coated paper-based nanosensor can visually detect TC in the concentration range of 0.1–30 µM. Moreover, the MTT assay indicates that the B,N-GQDs derived from natural products is an eco-friendly carbon material, which have little cytotoxicity to cells. Results obtained clearly indicate that B,N-GQDs synthesized from passion fruit juice can serve as a green nanosensing probe for practical detection of TC in aqueous and human serum samples, which can pave a gateway to provide an alternative to green synthesize the environmentally benign and biocompatible doped GQDs from naturally agricultural products for rapid and robotic detection of antibiotics and other organic metabolites in human serum and biological fluid samples. 

## Figures and Tables

**Table 1:** The analytical performance of various sensing nanomaterials toward TC detection.

Methods	Materials	LOD (nM)	Linear Range (μM)	Reference
Photoelectrochemical	Fe_2_O_3_ on Bi_2_WO_6_	300	0.01–25	[42]
Colorimetric aptasensor	THMS/AuNPs	0.27	3 × 10^−4^–10^−2^	[43]
Electrochemical	Au/C_3_N_4_	30	0.1–20 20–200	[44]
Electrochemical immunosensor	Bio-MEMS	1.2 pg mL^−1^	0.1–1000 pg mL^−1^	[19]
Ratiometric fluorescence	DPA-Ce-GMP-Eu	6.6	0.01–45	[45]
Ratiometric fluorescence	Carbon dot–Eu^3+^	11.6	0–7.8	[46]
Ratiometric fluorescence	Eu-GQDs	300	0–20	[28]
Fluorescence	MIPs-AF@SiO_2_	4.26	0–0.6	[47]
Fluorescence	Carbon dots	6,000	10–400	[27]
Fluorescence	B,N-GQDs	1 (PBS) 1.9 (urine) 2.2 (human serum)	0.04–14 0.06–14 0.06–14	This work

## Supplementary Materials

The following are available online at https://www.mdpi.com/2079-4991/10/9/1883/s1, Figure S1: (a) The full and (b) partial Raman spectrum of B,N-GQDs, and (c) the XRD pattern of B,N-GQDs on the Si substrate, Figure S2: (a) High resolution scanning electron microscopy (HRSEM) image, (b) energy dispersive spectroscopy (EDS) spectrum and elemental mapping of (c) C, (d), O (e), N and (f) B elements of B,N-GQDs, Figure S3: The (a) XPS deconvoluted C 1s and (b) O 1s peaks of N-GQD, Figure S4: The UV-visible spectra of B,N-GQDs, tetracycline (TC), and B,N-GQDs/TC, Figure S5: (a,b) The change in fluorescence of B,N-GQDs in the absence and the presence of TC (30 µM) under visible light and (c,d) under 365-nm UV light irradiation and in PBS solution, Figure S6: The linear relationship between fluorescence curve areas and absorbance for (a) pure N-GQDs, (b) B,N-GQDs, and (c) quinine sulfate standard, Figure S7: (a) The change in fluorescence emission spectra of B,N-GQDs at low TC concentration range of (0.06–14 nM) in urine and (b) human serum, Figure S8: The effect of pH on the fluorescence intensity of B,N-GQDs before and after the addition of 30 µM tetracycline. The pH is controlled at 3–10 in the presence of 0.1M PBS, Table S1: Elemental analysis of passion fruit juice, Table S2: Elemental weight percentage of elements in pure N-GQDs and B,N-GQDs estimated from survey scan of XPS, Table S3: Comparison of the quantum yield of 0-dimensional carbon-based nanomaterials and GQDs) synthesized from various natural products, Table S4: Zeta potential of B,N-GQDs, tetracycline (TC), B,N-GQDs/TC at various pHs.
