# Supplementary material for: Ultrasensitive Detection of Tetracycline Using Boron and Nitrogen Co-Doped Graphene Quantum Dots from Natural Carbon Source as the Paper-Based Nanosensing Probe in Difference Matrices
Source: Nanomaterials (Basel). 2020 Sep 20;10(9):1883. doi: 10.3390/nano10091883 (PMC7558855; doi:10.3390/nano10091883)
Supplement: Supplementary file 1 [file nanomaterials-10-01883-s001.pdf]

Supplementary Materials

Ultrasensitive detection of tetracycline using boron and nitrogen co-doped graphene quantum dots from natural carbon source as the paper-based nanosensing probe in difference matrices

Hai Linh Tran[1], Win Darmanto[2], and Ruey-an Doong[2,3*]

1. 101, Sec. 2, Kuang Fu Road, Department of Biomedical Engineering and Environmental Sciences, National Tsing Hua University, Hsinchu, 30013, Taiwan.
2. Department of Biology, Faculty of Science and Technology, Airlangga University, Surabaya, 60115, Indonesia.
3. 101, Sec. 2, Kuang Fu Road, Institute of Analytical and Environmental Science, National Tsing Hua University, Hsinchu, 30013, Taiwan.

Corresponding author: Ruey-An Doong (radoong@mx.nthu.edu.tw)

Table S1. Elemental analysis of passion fruit juice

Produce	Weight percentage of element (%)				
	C	O	N	H	S
Passion fruit juice	38.3	54.1	0.75	6.57	0.28

Table S2. Elemental weight percentage of elements in pure N-GQDs and B,N-GQDs estimated from survey scan of XPS.

**Materials**	**Weight percentage of element (%)**			
	C	O	N	B
N-GQDs	60.1	33.4	6.5	_[a]
B,N-GQDs	59.1	32.3	6.2	2.4

a: Not detected.

Table S3. Comparison of the quantum yield of 0-dimensional carbon-based nanomaterials and GQDs) synthesized from various natural products.

**Materials[a]**	**Precursors**	**Reaction Conditions**	**Quantum yield (%)**	**Reference**
CDs	Manilkara zapota fruits	100 °C, 60 min	5.2 – 7.9	[1]
CDs	Watermelon peel	220 °C, 2 h	7.1	[2]
CDs	Ocimum sanctum	180 °C, 4 h	9.3	[3]
CDs	Thymus vulgaris L	180 °C, 5 h	5.2	[4]
CDs	Pomelo peel	200 °C, 3 h	6.9	[5]
CDs	Aloe	180 °C, 11 h	10.4	[6]
P-CQDs	Pine wood	180 °C, 3 h	4.7	[7]
N, P- CQDs	Eleocharis dulcis	120 °C, 5 h	11.2	[8]
CDs	Sewage sludge	700 W, 30 min	21.7	[9]
CQDs	Bamboo tar	170 °C, 15 min	19.3	[10]
PEG-CDs	Gelatin	600 W, 10 min	34	[11]
CDs	Milk protein	30 min	18.7	[12]
**B,N-GQDs[b]**	**Passion fruit juice**	**170 °C, 20 min**	**50**	**This study**

a: CDs: carbon dots; CQDs: carbon quantum dots, GQDs: graphene quantum dots

b: B,N-GQDs

References

1. Bhamore, J.R.; Jha, S.; Park, T.J.; Kailasa, S.K. Green synthesis of multi-color emissive carbon dots from Manilkara zapota fruits for bioimaging of bacterial and fungal cells. *J. Photochem. Photobio. B* **2019,** 191, 150-155.
2. Zhou, J.; Sheng, Z.; Han, H.; Zou, M.; Li, C. Facile synthesis of fluorescent carbon dots using watermelon peel as a carbon source. *Mater. Lett.* **2012,** 66, 222-224.
3. Kumar, A.; Chowdhuri, A.R.; Laha, D.; Mahto, T.K.; Karmakar, P.; Sahu, S.K. Green synthesis of carbon dots from Ocimum sanctum for effective fluorescent sensing of Pb^{2+} ions and live cell imaging. *Sens. Actuators B Chem.* **2017,** 242, 679-686.
4. Bayat, A.; Masoum, S.; Hosseini, E.S. Natural plant precursor for the facile and eco-friendly synthesis of carbon nanodots with multifunctional aspects. *J. Mol. Liq.* **201**9, 281, 134-140.
5. Sahu, S.; Behera, B.; Maiti, T.K.; Mohapatra, S. Simple one-step synthesis of highly luminescent carbon dots from orange juice: application as excellent bio-imaging agents. *Chem. Commun.* **201**2, 48, 8835-8837.
6. Xu, H.; Yang, X.; Li, G.; Zhao, C.; Liao, X. Green synthesis of fluorescent carbon dots for selective detection of tartrazine in food samples. *J. Agric. Food Chem.* **2015,** 63, 6707-6714.
7. Zhao, S.; Song, X.; Chai, X.; Zhao, P.; He, H.; Liu, Z. Green production of fluorescent carbon quantum dots based on pine wood and its application in the detection of Fe^{3+}. *J. Clean Prod.* **2020,** 121561.
8. Bao, R.; Chen, Z.; Zhao, Z.; Sun, X.; Zhang, J.; Hou, L.; Yuan, C. Green and facile synthesis of nitrogen and phosphorus co-doped carbon quantum dots towards fluorescent ink and sensing applications. *Nanomaterials* **2018,** 8, 386.

9. Hu, Y.; Gao, Z. Sewage sludge in microwave oven: A sustainable synthetic approach toward carbon dots for fluorescent sensing of para-nitrophenol. *J. Hazard Mater.* **2019,** 121048.

10. Liang, Q.; Wang, Y.; Lin, F.; Jiang, M.; Li, P.; Huang, B. A facile microwave-hydrothermal synthesis of fluorescent carbon quantum dots from bamboo tar and their application. *Anal Methods* **2017**, 9, 3675-3681.

11. Arsalani, N.; Nezhad-Mokhtari, P.; Jabbari, E. Microwave-assisted and one-step synthesis of PEG passivated fluorescent carbon dots from gelatin as an efficient nanocarrier for methotrexate delivery. *Artif. Cells Nanomed Biotechno.* **2019,** 47, 540-547.

12. Bajpai, S.; D'Souza, A.; Suhail, B.J.I.N.L. Blue light-emitting carbon dots (CDs) from a milk protein and their interaction with Spinacia oleracea leaf cells. *Int. Nano Lett.* **2019**, 1-10.

Table S4. Zeta potential of B,N-CDs, tetracycline (TC), B,N-CDs/TC at various pHs.

pH value	Zeta potential (mV)		
	B,N-CDs	Tetracycline (TC)	B,N-CDs/TC
pH 3	-11	-3.34	-3.32
pH 4	-4.89	-3.74	-2.28
pH 5	-14.9	-6.26	-3.45
pH 6	-19.5	-14.1	-2.91
pH 7	-0.86	-24.1	-3.23
pH 8	-2.51	-2.13	-3.66
pH 9	-4.16	-9.99	-1.02
pH 10	-6.66	-4.95	-4.74

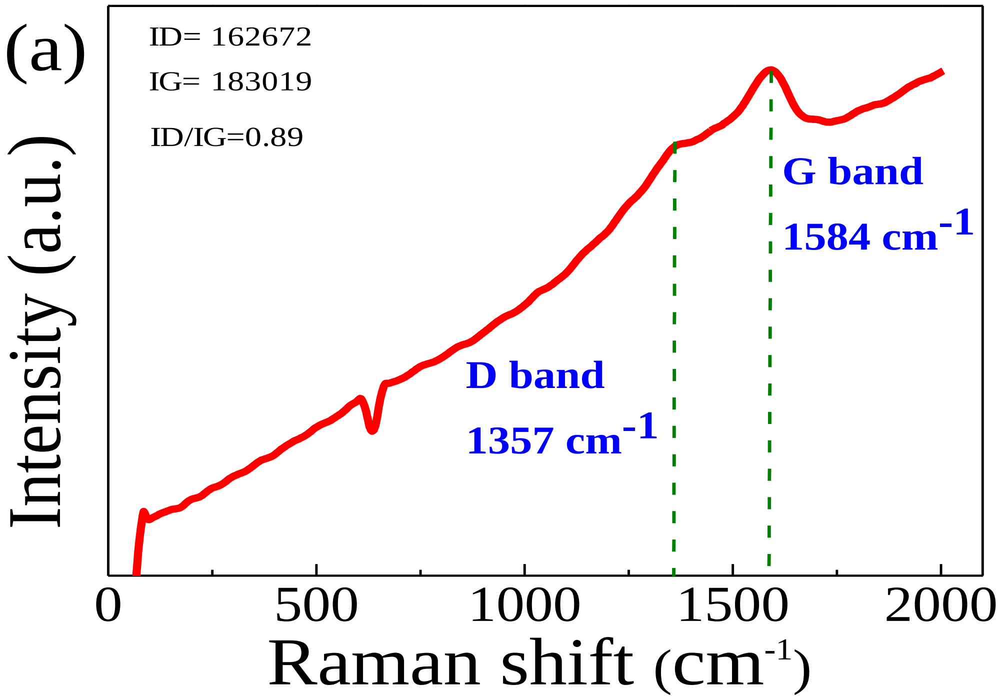
(a)
ID= 162672
IG= 183019
ID/IG=0.89
G band
1584 cm-1
D band
1357 cm-1
Intensity (a.u.)
0
500
1000
1500
2000
Raman shift (cm-1)

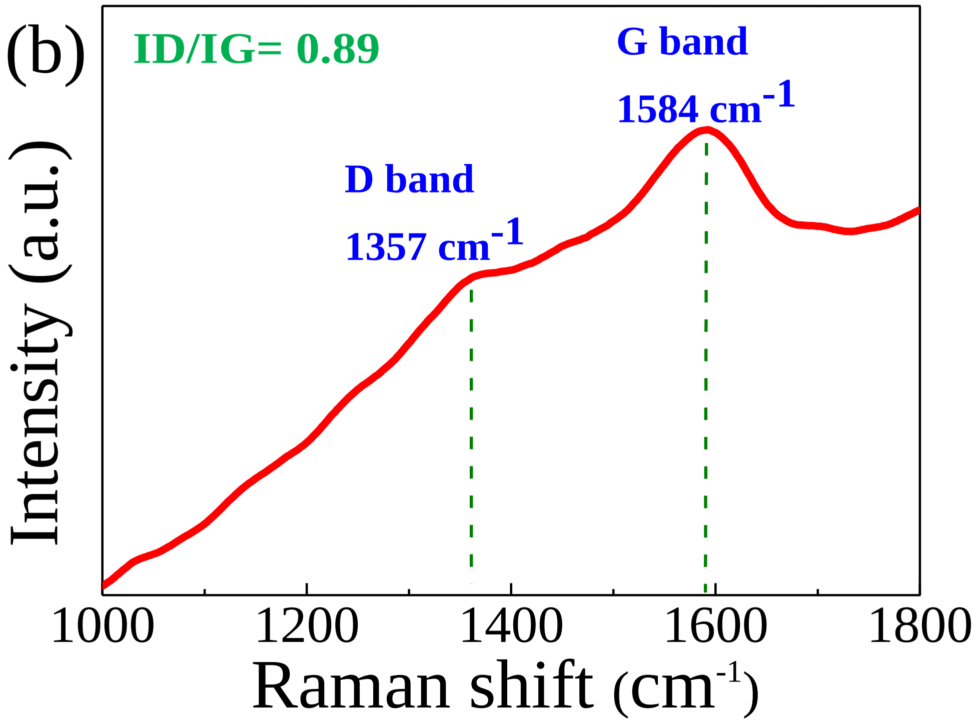
(b)
ID/IG= 0.89
G band
1584 cm-1
D band
1357 cm-1
Intensity (a.u.)
1000
1200
1400
1600
1800
Raman shift (cm-1)

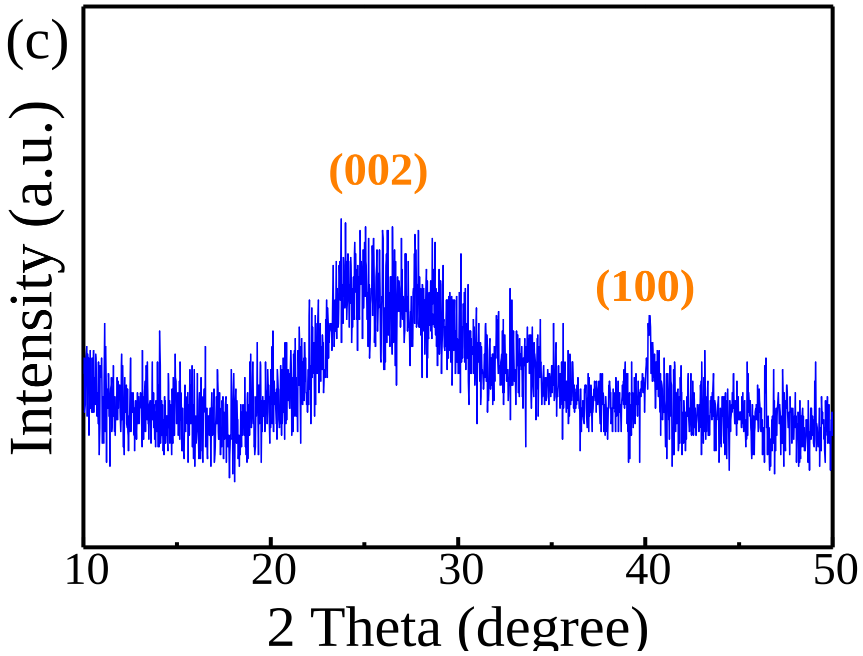

Fig. S1. (a) The full and (b) partial Raman spectrum of B,N-GQDs, and (c) the XRD pattern of B,N-GQDs on the Si substrate.

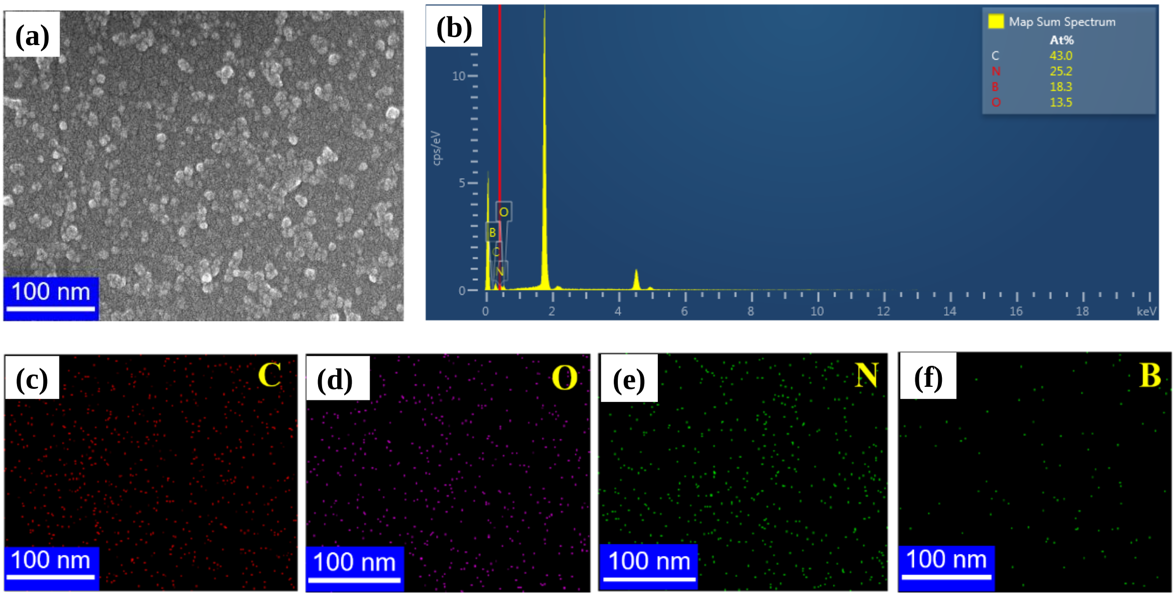

Fig. S2. (a) High resolution scanning electron microscopy (HRSEM) image, (b) energy dispersive spectroscopy (EDS) spectrum and elemental mapping of (c) C, (d), O (e), N and (f) B elements of B,N-GQDs.

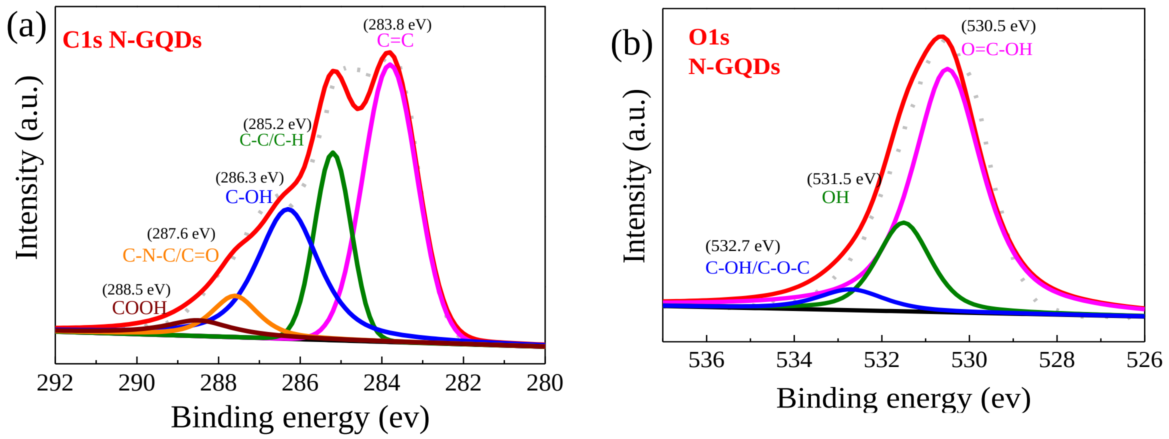

Fig. S3. The (a) XPS deconvoluted C 1s and (b) O 1s peaks of N-GQD.

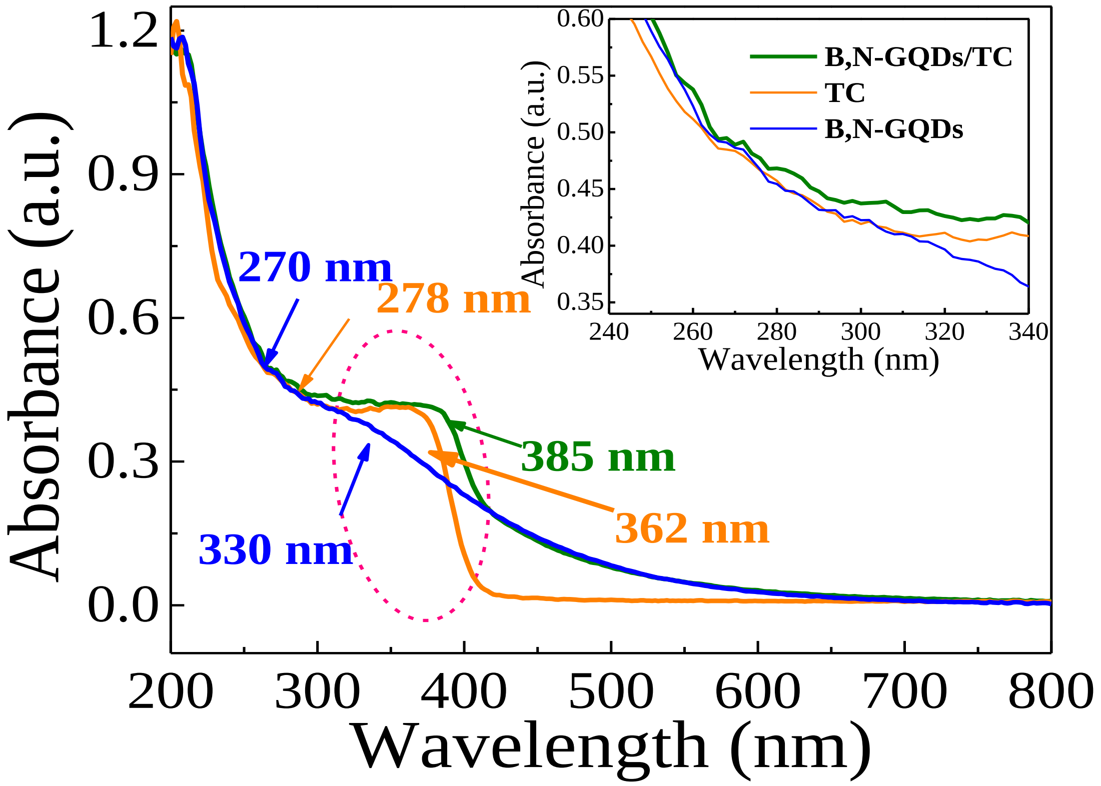

Fig. S4. The UV-visible spectra of B,N-GQDs, tetracycline (TC), and B,N-GQDs/TC.

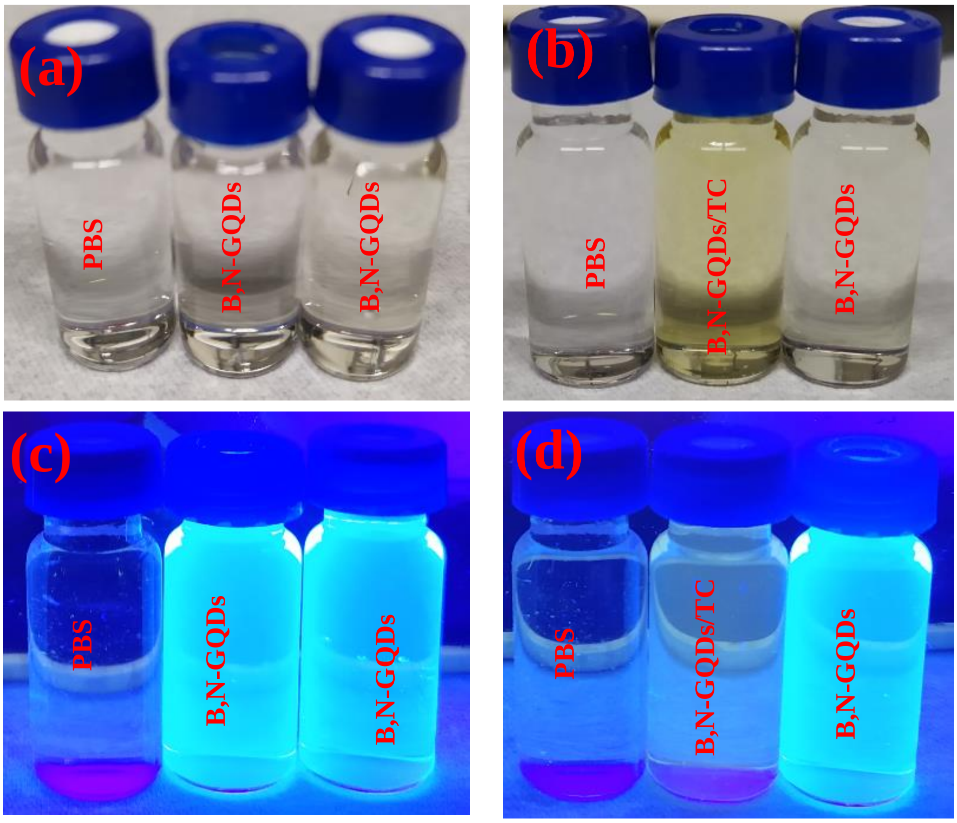

Fig. S5. (a) (b) The change in fluorescence of B,N-GQDs in the absence and the presence of TC (30 μM) under visible light and (c) (d) under 365-nm UV light irradiation and in PBS solution.

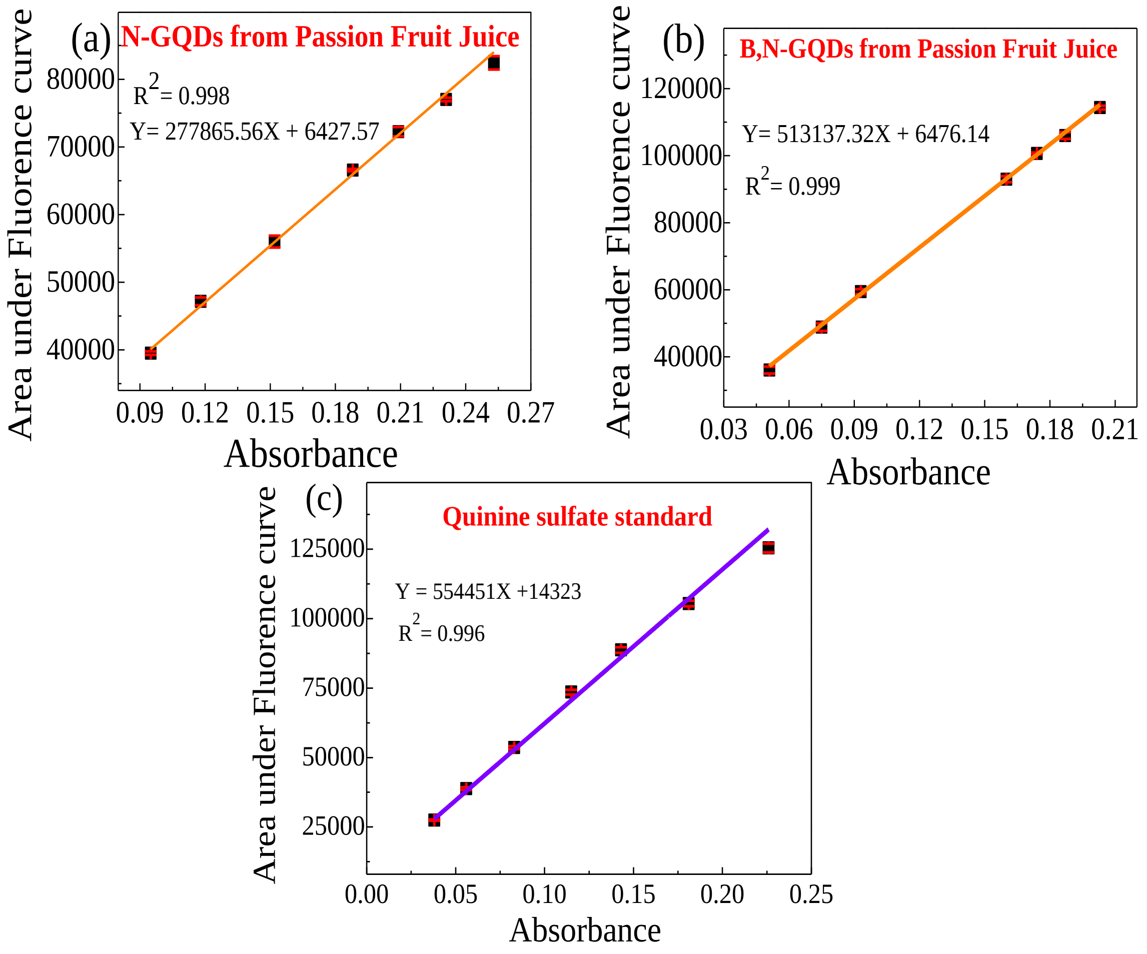

Fig. S6. The linear relationship between fluorescence curve areas and absorbance for (a) pure N-GQDs, (b) B,N-GQDs, and (c) quinine sulfate standard.

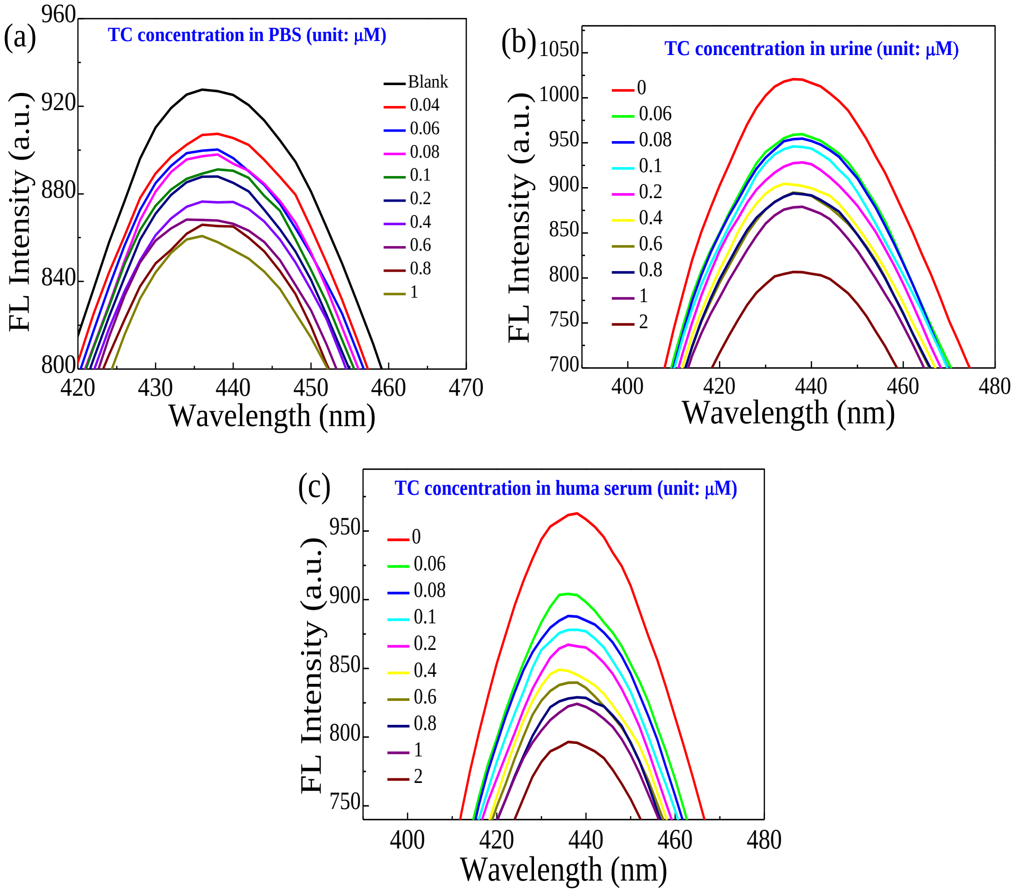

Fig. S7. (a) The change in fluorescence emission spectra of B,N-GQDs at low TC concentration range of (0.06~14 nM) in urine and (b) human serum.

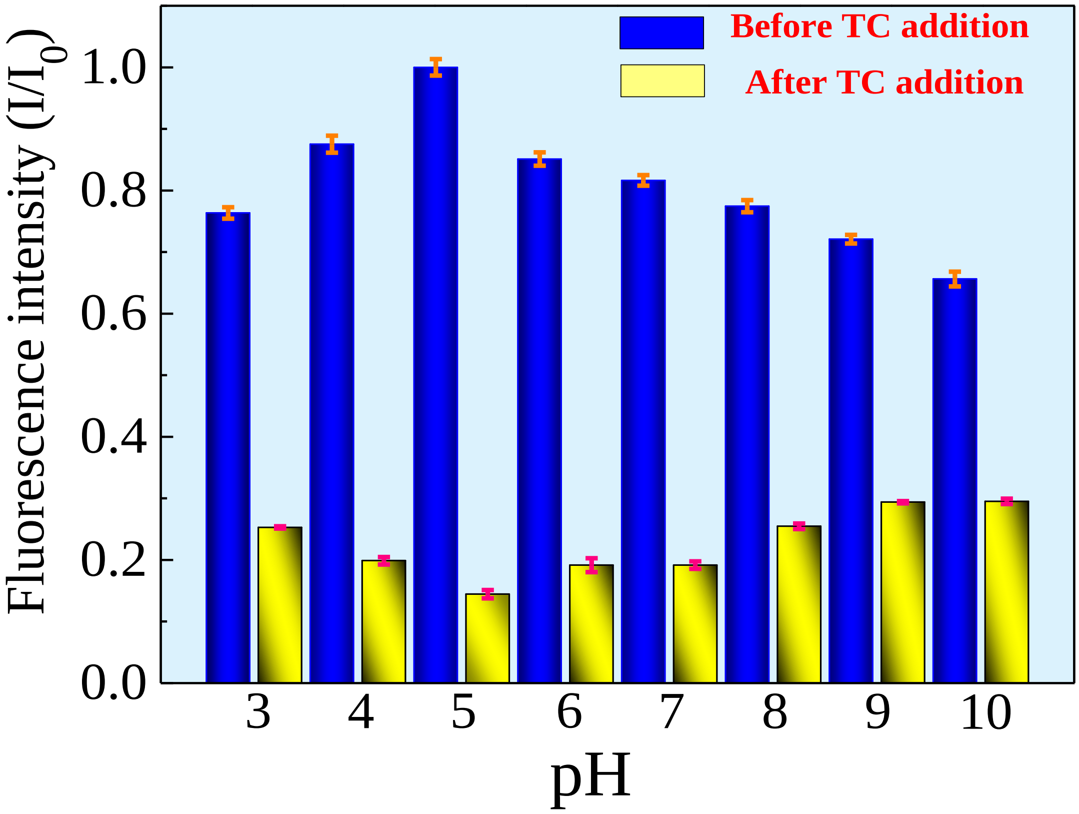

Fig. S8. The effect of pH on the fluorescence intensity of B,N-GQDs before and after the addition of 30 μM tetracycline. The pH is controlled at 3 – 10 in the presence of 0.1M PBS.
